# 132 Behavioural change for Parkinson’s Disease: A randomised controlled feasibility study to promote physical activity and exercise adherence among people with Parkinson’s Disease

**DOI:** 10.1093/eurpub/ckae114.045

**Published:** 2024-09-26

**Authors:** Leanne Ahern, Suzanne Timmons, Sarah E Lamb, Ruth McCullagh

**Affiliations:** University College Cork, Cork, Ireland; University College Cork, Cork, Ireland; University of Exeter, Exeter, United Kingdom; University College Cork, Cork, Ireland

## Abstract

**Background:**

Physical activity and exercise can improve health, but many people with Parkinson’s (PwP) have trouble achieving the recommended dosage. Our novel behavioural change intervention is informed by a recent literature review, qualitative study, and patient-public input. This is designed to complement an existing exercise programme, to improve exercise adherence and physical activity.

**Objective:**

To evaluate recruitment, data collection procedures, acceptability of the program, resources required for the study, and trends in the range and variability in measures of physical activity, function, and self-efficacy in PwP.

**Methods:**

A parallel-arm, single blinded, randomised feasibility study. Participants (Hoehn and Yahr stage 1-3) were recruited from a physiotherapy primary-care waiting list and randomly allocated (stratified by sex) to the intervention (PEEP+BC) or the control group (PEEP). Both groups received usual care; a 12-week program of weekly multidisciplinary education sessions, supervised exercise, and prescribed home exercises. The intervention group received additional behavioural change techniques, targeting behaviour regulation, belief about capabilities and social influences. Enrolment, attendance and exercise adherence rates, and negative events were recorded. Physical activity, function, falls, and exercise self-efficacy were measured. We used surveys to examine acceptability and satisfaction and exit interviews to further explore the experience of the behavioural change techniques.

**Results:**

Twenty-six people were screened; sixteen people participated. Attendance rates (PEEP+BC; 86%, PEEP; 75%), and home adherence (PEEP+BC, 83%; PEEP, 70%) were high. No negative events were detected. Time resources were estimated as acceptable [2-2.5 hours per week] and pre-post assessments took an average of 30 minutes. Accelerometery data, self-efficacy and exercise endurance were most sensitive to changes. The acceptability of behavioural change techniques was mixed and suggests that profiling preferences could lead to a more individualised intervention. All participants enjoyed the group dynamic and gaining knowledge and skills was frequently perceived as important.

**Conclusion:**

Findings suggest that the study of PEEP+BC is feasible and acceptable in PwP. Preference profiling could lead to a more tailored behavioural change intervention. Future research is required to compare effectiveness in a larger sample.

**Trial Registration:**

ClincialTrials.gov NCT06192628

**Support/Funding Source:**

Irish Research Council Enterprise Partnership Scheme (in conjunction with the Parkinson’s Association of Ireland).

